# : Short-term effects of exposure to particulate matter on hospital admissions for asthma and chronic obstructive pulmonary disease

**DOI:** 10.1097/MD.0000000000030165

**Published:** 2022-09-02

**Authors:** Chang Hoon Han, Haeyong Pak, Jung Mo Lee, Jae Ho Chung

**Affiliations:** a Department of Internal Medicine, National Health Insurance Service Ilsan Hospital, Goyang, Republic of Korea; b Research and Analysis Team, National Health Insurance Service Ilsan Hospital, Goyang, Republic of Korea; c Department of Internal Medicine, International St. Mary`s Hospital, Catholic Kwandong University College of Medicine, Incheon, Republic of Korea.

**Keywords:** air pollution, asthma, chronic obstructive pulmonary disease, South Korea

## Abstract

We investigated the effects of particulate matter (PM) factors on hospitalization rates for asthma and chronic obstructive pulmonary disease (COPD).

We obtained data on pollutants—PM_10_, PM_2.5_—in Seoul, South Korea. We also investigated data for asthma and COPD exacerbation that required hospitalization from 2006 to 2016. We used a time-stratified case-crossover design and generalized additive models with log transformation to assess adjusted risk, and conditional logistic regression was performed to analyze these data.

Our study showed that PM_10_ and PM_2.5_, on different best lag days, were associated with increased risks of COPD or asthma hospitalization. The odds ratios (ORs) for each per-unit increase in PM_10_ and PM_2.5_ were higher in patients with male asthma (PM_10_: OR, 1.012; 95% confidence interval [CI], 1.008–1.016 and PM_2.5_: OR, 1.015; 95% CI, 1008–1.023), preschool asthma (PM_10_: OR, 1.015; 95% CI, 1.006–1.015 and PM_2.5_: OR, 1.015; 95% CI, 1.009–1.024), male COPD (PM_10_: OR, 1.012; 95% CI, 1.005–1.019 and PM_2.5_: OR, 1.013; 95% CI, 1.000–1.026), and senior COPD (PM_10_: OR, 1.016; 95% CI, 1.008–1.024 and PM_2.5_: OR, 1.022; 95% CI, 1.007–1.036).

Increasing PM levels increased hospitalizations for asthma and COPD. Additionally, the consequences may be different according to age and sex, and PM_2.5_ may have a more significant effect on airway disease patients than PM_10_.

## 1. Introduction

Harmful air pollutants are mainly caused by particulate matter (PM) generated during the combustion of fossil fuels. PM less than 10 μm in diameter (PM_10_) in the air penetrates alveoli in the lungs and causes increased mortality.^[1]^ PM_10_ also induces diseases, such as respiratory and cardiovascular diseases.^[2,3]^ PM less than 2.5 μm in diameter (PM_2.5_) is known to represent an even greater health hazard than PM_10_. PM_2.5_ can penetrate deeper into the respiratory tract than PM_10_ and, considering the anatomical structure of the lungs and the flow of air in the respiratory airway, shows greater health effects such as inflammatory response of the alveoli.^[4]^ The chemical composition of PM_2.5_ comprises more harmful substances, such as fossil fuels and industrial emissions, than that of PM_10_.^[5]^ Many studies have established an association between PM and respiratory diseases. A study reported that lung function in a large population exposed to air pollutants was 4.9 times lower than that of a nonexposed population.^[6]^ Studies have also reported that chronic obstructive pulmonary disease (COPD) development is associated with decreased lung function related to long-term exposure to air pollutants.^[7]^ Over 5 years, an increase in PM_10_ concentration of 7 μg/m^3^ caused a 5.1% decrease in forced expiratory vital capacity in 1 second, a 3.7% decrease in forced vital capacity, and a 33% increase in the incidence of COPD.^[8]^ An increase of PM_10_ by 10 μg/m^3^ was reported to exacerbate asthma by 29%; this percentage reflects the increase in emergency department (ED) visits resulting in hospitalization for asthma exacerbation.^[9,10]^ In addition, there have been some studies on the relationship between PM_10_ and PM_2.5_, and respiratory diseases in Korea. Based on the concentration of PM_2.5_, in Chuncheon between 2006 and 2012, visits to hospitals due to exacerbation of COPD increased as PM_2.5_ concentration increased.^[11]^ A study based on PM data from Busan from 2007 to 2010 confirmed that hospitalization due to respiratory diseases (acute bronchitis, allergic rhinitis, and asthma) increased when the concentration of PM increased.^[12]^ A study of the effects of PM_10_ on lung function in Korean junior high school students demonstrated an association between increased PM_10_ levels and decreased lung function.^[13]^ However, there have been no studies regarding the effects of PM_10_, and PM_2.5_ affect on asthma and COPD hospitalizations. An updated study on the effect of PM on asthma and COPD hospitalizations is important for effective actions in South Korea. Therefore, we investigated the data from the Korean National Health Insurance records and assessed the effects of PM_10_ and PM_2.5_ for asthma and COPD hospitalization in Seoul, South Korea.

## 2. Methods

Environmental monitoring data on PM_10_ and PM_2.5_ were investigated hourly at each monitoring station from 2006 to 2016. For PM, air pollution measurement data from 2006 to 2016 of the Seoul City Institute of Health and Environment was used. Seoul City operates 27 air pollution automatic measurement stations and measures the concentrations of PM_10_ and PM_2.5_ every hour. Hourly measurement data were reprocessed to reflect an average daily concentration. The meteorological data provided by the Korea Meteorological Agency using an Automated Synoptic Observation System were measured in 3-hour units for each meteorological element and reprocessed to a daily average. PM data were collected, and meteorological and hourly data on 24 hours mean temperature (°C), humidity (%), and atmospheric pressure (hPa) at sea level were obtained from the Korea Meteorological Administration.

### 2.1. Study populations

We obtained medical record data from 2006 to 2016 from the National Health Information Database of the National Health Insurance Service. National Health Insurance Service Ilsan Hospital 2019-04-020 IRB protocol number that was assigned to our study by the Institutional Review Board.

Patients with asthma or COPD exacerbation who needed hospitalization were included. For subgroup analyses, our study categorized asthma patients as preschoolers (0–5 years old), childhood (6–17 years old), adults (18–64 years old), or seniors (≥ 65 years old), and COPD patients were classified as adults (40–64 years old) or seniors (≥ 65 years old).

### 2.2. Study design

Our study used a time-stratified case-crossover design^[14]^ to investigate the acute effects of daily PM levels on asthma or COPD patient hospitalizations. This case-crossover study design required exposure data for only cases, as it was a special type of case-control study in which each case served as its own control. This design is used in environmental epidemiologic studies, and one advantage is that confounding effects due to individual characteristics that do not vary by age, gender, physical condition, and time are removed. A generalized additive model was used to quantitatively assess the health effects of air pollution, and disturbance variables such as long-term trends and day-of-week effects and weather factors such as temperature, humidity, and barometric pressure were corrected. The final analysis model for the effects of respiratory-related hospitalization is as follows;


$$\begin{array}{l} \text{ln}\;E\left[Y\right]=\beta 0+\beta 1\left(Pollutant\right)+D\left(Day\;of\;week\right)+S1\left(Number\;of\;days\right)+S2\left(Temperature\right)+S3\left(Humidity\right)+S4\left(Press\right) \end{array}$$

Here, E [Y] is the number of daily respiratory disease-related inpatients, and D (day of week) is an applied variable number that reflects weekday, holiday, Saturday, and Sunday to correct for variability in days of the week and weekday/holiday effects. Temperature, relative humidity, and barometric pressure were corrected with the locally estimated scatterplot smoothing function for each variable to estimate the relative risk ratio. We defined an existing tolerance standard of PM as the threshold and applied the Korean and World Health Organization (WHO) standards for PM_10_ and PM_2.5._ The value of Akaike information criterion was used as a model suitability criterion to find the optimal threshold value and to evaluate the health effects of PM, and the model with the smallest value of Akaike information criterion was selected. In the health effect assessment of PM_10_ and PM_2.5_, the lag effect on the day of occurrence and the previous 14 days was also evaluated in consideration of possible health effects.

### 2.3. Data analyses

A time-stratified, case-crossover, generalized additive model was used to evaluate the relationships between asthma and COPD hospitalizations and concentrations of outdoor PM_10_ and PM_2.5_ on the current day, single-lag days, and multi-lag days, respectively.^[15,16]^ The effect of the concentration of each PM was included in the single-day models for only a specific day (lag 0, lag 1, lag 2, etc.). In the multi-lag day models, we obtained the combined effect of multiple days (e.g., from lag 0 to lag 7). Lag time 0 (i.e., lag 0) was defined as same-day exposure to PM_10_ and PM_2.5_. For example, lag 0 means that the effect of PM was observed on the current day, lag 1 means that the effect was observed on the previous day, and lag 7 means that the effect was observed 7 days before. The lags between consecutive days were calculated as the intervals. This lag time was evaluated because some time is needed for PM to affect health events. In addition to PM exposure, our study was also adjusted to include data for mean temperature, humidity, and atmospheric pressure at sea level.

In all models, relative risk was estimated on the basis of an increase of 1 unit of the PM, with correction for temperature, relative humidity, and atmospheric pressure at sea level. *P* < .05 was considered statistically significant. All data were analyzed with R version 3.6.3 (R Foundation for Statistical Computing, Vienna, Austria, http://cran.r-project.org).

## 3. Results

### 3.1. Baseline characteristics of study population

Table 1 shows the general characteristics of asthma (n = 167,260) and COPD (n = 156,786) exacerbations requiring hospitalization due to partly PM.

**Table 1:** General characteristics of patients.

Asthma (n = 167,260)	
Sex	
Male	90,823 (54.3)
Female	76,437 (45.7)
Age	
Preschool (0–5 yr)	59,377 (35.5)
Adolescents (6–17 yr)	19,203 (11.5)
Adult (18–64 yr)	33,485 (20.0)
Senior (65 yr)	55,195 (33.3)
COPD (n = 156,786)	
Sex	
Male	91,550 (58.4)
Female	65,236 (41.6)
Age	
Adult (40–64 yr old)	37,629 (24.0)
Senior (65 yr old)	119,158 (76.0)

COPD = chronic obstructive pulmonary disease.

### 3.2. The results of air pollutants

Table 2 showed the 24 hours mean concentrations of PM_10_ and PM_2.5_. Table 3 shows the correlations between the PM and meteorological variables. PM_10_ and PM_2.5_ concentrations were strongly correlated (*r* = 0.772; *P* < .001).

**Table 2:** Summary statistics of particulate matter and meteorological variables.

	Mean ± SD	Minimum		Percentile		Maximum
			25	50	75	
PM_10_ (µg/m^3^)	49.9 ± 33.8	5.0	31.0	44.0	61.0	860.0
PM_2.5_ (µg/m^3^)	24.4 ± 14.3	3.0	14.5	21.7	30.3	215.1
Temperature (°C)	12.9 ± 10.6	–14.5	3.9	14.4	22.5	31.8
Humidity (%)	60.4 ± 14.9	19.9	49.3	60.5	71.0	99.8
Pressure (hPa)	1016.1 ± 8.2	990.8	1009.6	1016.4	1022.6	1038.1

PM = particulate matter, SD = standard deviation.

**Table 3:** Spearman’s correlation between particulate matter and meteorological variables.

	PM_10_	PM_2.5_	Temperature (°C)	Humidity (%)	Pressure (hPa)
PM_10_ (µg/m^3^)	1.000	0.772	–0.176	–0.0989	0.128
*P*		<.0001	<.0001	<.0001	<.0001
PM_2.5_ (µg/m^3^)		1.000	–0.135	0.030	0.1336
*P*			<.0001	.0593	<.0001
Temperature (°C)			1.000	0.389	–0.775
*P*				<.0001	<.0001
Humidity (%)				1.000	–0.494
*P*					<.0001
Pressure (hPa)					1.000

PM = particulate matter.

**Figure 1.**
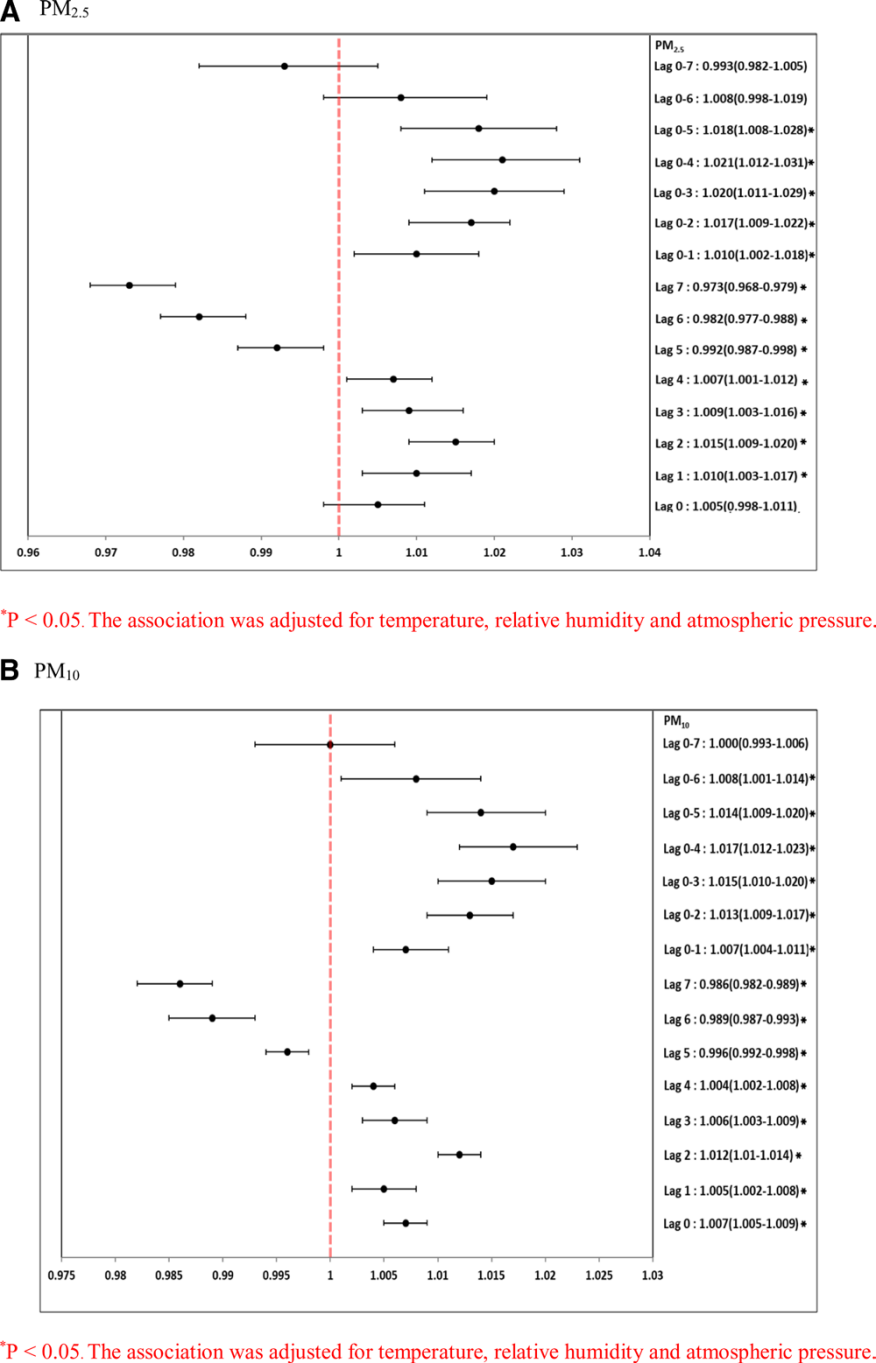
Forest-plot graphs of association between PM (A) PM_2.5_ and PM_10_ (per-unit increase in different lag days) for asthma hospitalizations. *P < .05. The association was adjusted for temperature, relative humidity and atmospheric pressure. PM = particulate matter.

**Figure 2.**
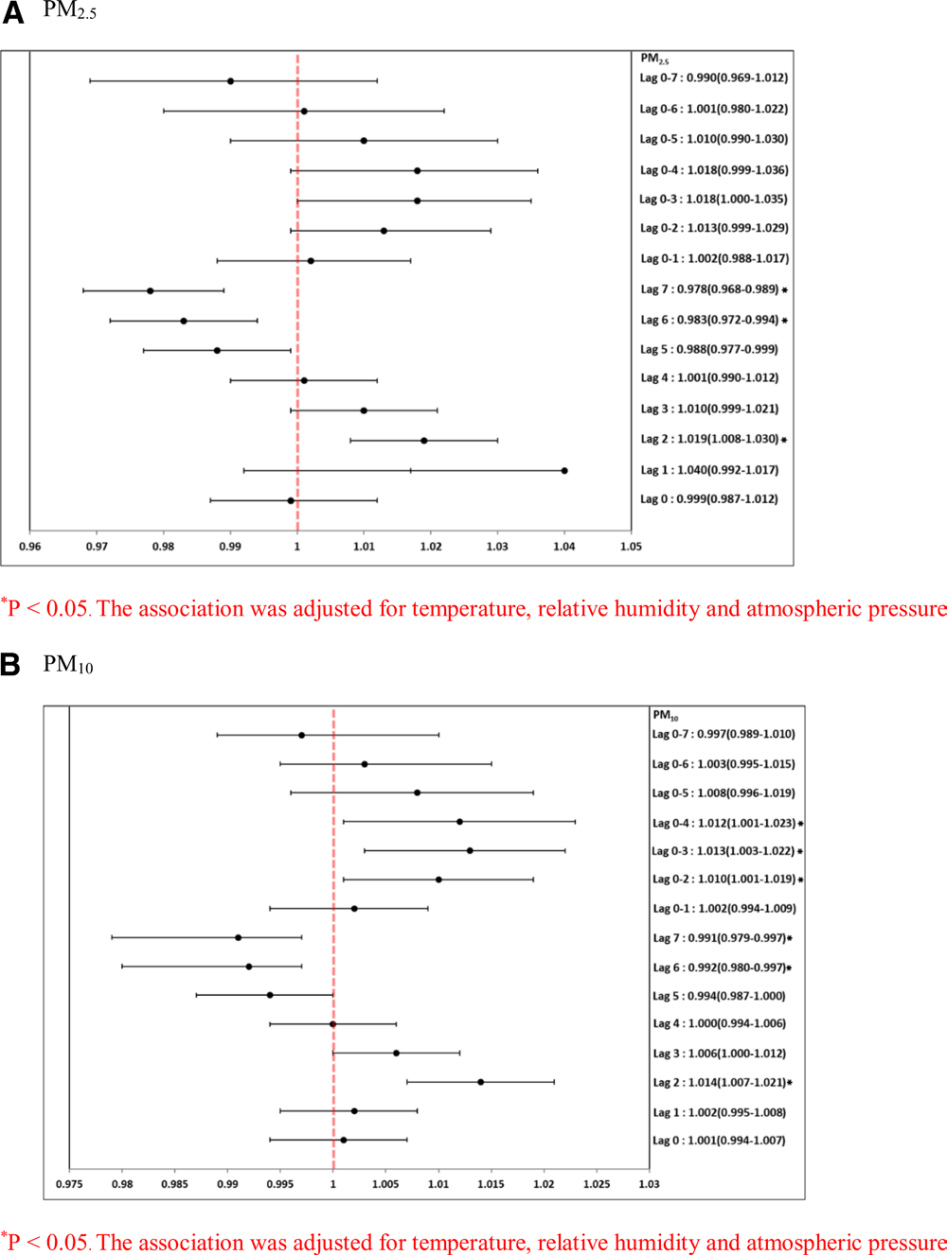
Forest-plot graphs of association between PM (A) PM_2.5_ and PM_10_ (per-unit increase in different lag days) for COPD hospitalizations. *P < .05. The association was adjusted for temperature, relative humidity and atmospheric pressure. COPD = chronic obstructive pulmonary disease, PM = particulate matter.

Table 4 and Figure 1 summarize the results of the single-pollutant model for hospital admission due to asthma, after controlling for meteorological variables. Per-unit increases in the concentrations of PM_10_ and PM_2.5_ were associated with an increased risk of asthma hospitalization on different best lag days. These per-unit increases in PM_10_ were significantly positively associated with asthma hospitalization on lags 0, 1, 2, 3, 4, 0–1, 0–2, 0–3, 0–4, 0–5, 0–6, and 0–7. Per-unit increases in PM_2.5_ were significantly positively associated with asthma hospitalization on lags 0, 1, 2, 3, 4, 0–1, 0–2, 0–3, 0–4, and 0–5. Per-unit increases in PM_10_ were significantly positively associated with asthma hospitalization on lags 1, 2, 3, 4, 0–2, 0–3, 0–4, 0–5, and 0–6. Table 5 and Figure 2 summarize the results of the single-pollutant model for COPD hospitalization after adjusting for meteorological variables. Per-unit increases in concentrations of PM_10_ and PM_2.5_ were associated with an increased risk of COPD hospitalization on different best lag days. Per-unit increases in PM_10_ were significantly positively associated with COPD hospitalization on lags 2, 3, 0–2, 0–3, and 0–4. Per-unit increases in PM_2.5_, were significantly positively associated with COPD hospitalization on lag 2 and lag 0–3.

**Table 4:** Association between particulate matter (per-unit increase in different lag days) for asthma hospitalizations: single-pollutant model.

Lag day	PM_10_	PM_2.5_
0	1.007 (1.003–1.009)*	1.005 (0.998–1.011)
1	1.005 (1.002–1.008)*	1.010 (1.003–1.017)*
2	1.012 (1.007–1.014)*	1.015 (1.009–1.020)*
3	1.006 (1.003–1.009)*	1.009 (1.003–1.016)*
4	1.004 (1.002–1.008)*	1.007 (1.001–1.012)*
5	0.996 (0.992–0.998)*	0.992 (0.987–0.998)*
6	0.989 (0.987–0.993)*	0.982 (0.977–0.988)*
7	0.986 (0.982–0.989)*	0.973 (0.968–0.979)*
0–1	1.007 (1.004–1.011)*	1.010 (1.002–1.018)*
0–2	1.013 (1.009–1.017)*	1.017 (1.009–1.022)*
0–3	1.015 (1.010–1.020)*	1.020 (1.011–1.029)*
0–4	1.017 (1.012–1.023)*	1.021 (1.012–1.031)*
0–5	1.014 (1.009–1.020)*	1.018 (1.008–1.028)*
0–6	1.008 (1.001–1.014)*	1.008 (0.998–1.019)
0–7	1.000 (0.993–1.006)	0.993 (0.982–1.005)

PM = particulate matter.

* *P* < .05. The association was adjusted for temperature, relative humidity and atmospheric pressure.

**Table 5:** Association between particulate matter (per-unit increase in different lag days) for COPD hospitalizations: single-pollutant model.

Lag day	PM_10_	PM_2.5_
0	1.001 (0.994–1.007)	0.999 (0.987–1.012)
1	1.002 (0.995–1.008)	1.040 (0.992–1.017)
2	1.014 (1.007–1.021)*	1.019 (1.008–1.030)*
3	1.006 (1.000–1.012)*	1.010 (0.999–1.021)
4	1.000 (0.994–1.006)	1.001 (0.990–1.012)
5	0.994 (0.987–1.000)	0.988 (0.977–0.999)
6	0.992 (0.980–0.997)	0.983 (0.972–0.994)*
7	0.991 (0.979–0.997)	0.978 (0.968–0.989)*
0–1	1.002 (0.994–1.009)	1.002 (0.988–1.017)
0–2	1.010 (1.001–1.019)*	1.013 (0.999–1.029)
0–3	1.013 (1.003–1.022)*	1.018 (1.000–1.035)
0–4	1.012 (1.001–1.023)*	1.018 (0.999–1.036)
0–5	1.008 (0.996–1.019)	1.010 (0.990–1.030)
0–6	1.003 (0.995–1.015)	1.001 (0.980–1.022)
0–7	0.997 (0.989–1.010)	0.990 (0.969–1.012)

COPD = chronic obstructive pulmonary disease, PM = particulate matter.

* *P* < .05. The association was adjusted for temperature, relative humidity and atmospheric pressure.

### 3.3. The correlation among air pollutants

**Figure 3.**
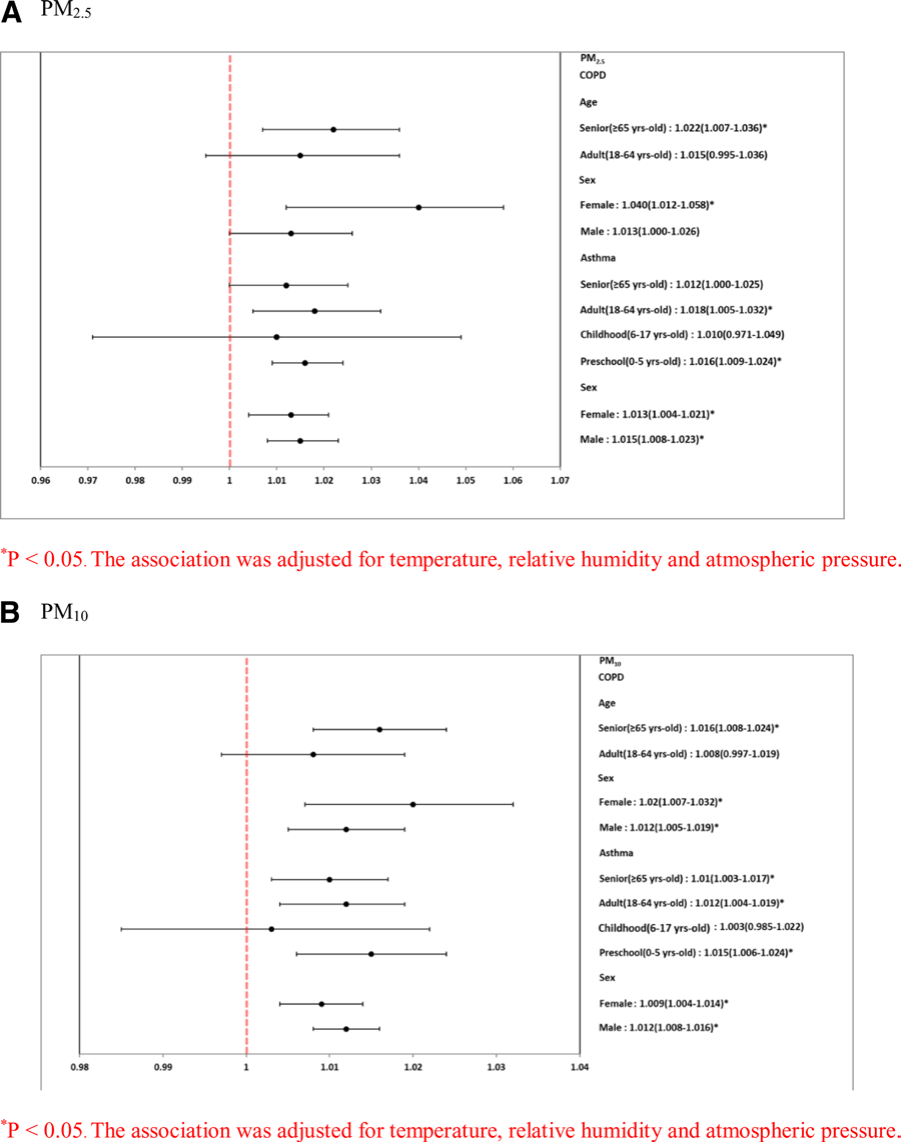
Forest-plot graphs of association between PM (A) PM_2.5_ and PM_10_ (per-unit increase in different lag days) for COPD for COPD and asthma hospitalizations. *P < .05. The association was adjusted for temperature, relative humidity and atmospheric pressure. COPD = chronic obstructive pulmonary disease, PM = particulate matter.

The effects of PM on the reasons for hospitalization, sex, and age are shown in Table 6 and Figure 3. The odds ratios (ORs) for each per-unit increase in PM_10_ and PM_2.5_ were higher in male patients with asthma (PM_10_: OR, 1.012; 95% confidence interval [CI], 1.008–1.016 and PM_2.5_: OR, 1.015; 95% CI, 1008–1.023), preschool asthma (0–5 years old) (PM_10_: OR, 1.015; 95% CI, 1.006–1.015 and PM_2.5_: OR, 1.015; 95% CI, 1.009–1.024), male COPD (PM_10_: OR, 1.012; 95% CI, 1.005–1.019 and PM_2.5_: OR, 1.013; 95% CI, 1.000–1.026), and senior COPD (≥ 65 years old) (PM_10_: OR, 1.016; 95% CI, 1.008–1.024 and PM_2.5_: OR, 1.022; 95% CI, 1.007–1.036).

**Table 6:** Association between particulate matter (per-unit increase in different best lag days) for asthma and COPD hospitalizations.

	PM_10_	PM_2.5_
Asthma		
Sex		
Male	1.012 (1.008–1.016)*	1.015 (1.008–1.023)*
Female	1.009 (1.004–1.014)*	1.013 (1.004–1.021)*
Age		
Preschool (0–5 yr old)	1.015 (1.006–1.015)*	1.016 (1.009–1.024)*
Childhood (6–17 yr old)	1.003 (0.985–1.022)	1.010 (0.971–1.049)
Adult (18–64 yr old)	1.012 (1.004–1.019)*	1.018 (1.005–1.032)*
Senior (≥65 yr old)	1.010 (1.003–1.017)*	1.012 (1.000–1.025)
COPD		
Sex		
Male	1.012 (1.005–1.019)*	1.013 (1.000–1.026)
Female	1.020 (1.007–1.032)*	1.040 (1.012–1.058)*
Age		
Adult (40–64 yr old)	1.008 (1.000–1.019)	1.015 (0.995–1.036)
Senior (≥65 yr old)	1.016 (1.008–1.024)*	1.022 (1.007–1.036)*

COPD = chronic obstructive pulmonary disease, PM = particulate matter.

* *P* < .05. The association was adjusted for temperature, relative humidity and atmospheric pressure.

## 4. Discussion

Our study showed that exposure to PM exacerbated the risk of asthma and COPD hospitalization. Our subgroup analyses of sex- and age-specific effects on the association between exposure to PM and risk for asthma and COPD hospitalization showed that male asthma, preschool asthma, male COPD, and senior COPD patients had a higher risk of asthma and COPD hospitalizations.

The size of PM plays an essential role in determining the deposition site in the lungs.^[17]^ Our subgroup analysis showed that PM_2.5_ may have a more significant effect on asthma and COPD hospitalization than PM_10._ These findings may be because while the coarse fraction (PM_2.5–10_) can penetrate the airways, the fine fraction (PM_1–2.5_) may be deposited in the lungs, particularly in the alveoli, and may pass into systemic circulation.^[18]^ There are also differences in individuals’ susceptibility to PM. According to a number of meta-analyses,^[15,16,19]^ PM was associated with increased asthma hospitalization and worsening of asthma symptoms. Many epidemiological studies have demonstrated the association between PM and cardiovascular diseases^[20,21]^ and respiratory diseases.^[14,22]^ However, few studies have focused on the PM_2.5_ constituents and hospitalization for respiratory diseases. PM may affect respiratory health by producing reactive oxygen species in the airway tract. Reactive oxygen species caused by PM might cause respiratory epithelial damage and inflammatory pathophysiology, thereby exacerbating asthma and COPD.^[23]^

Our study showed that PM exacerbates the risk of asthma and COPD hospitalizations, which is consistent with previous studies.^[24–26]^ Hwang et al^[24]^ showed that PM_2.5_ is associated with asthma-related ED visits. Other studies have shown a positive association between PM_2.5_ and COPD-related ED visits,^[25,26]^ but still others did not show a positive association.^[27, 28]^ These inconsistent findings may be due to differences in lag days collected in previous studies. Gao et al^[14]^ found a strong association between PM_2.5_ and COPD-related hospitalization on lags 0 to 7 (average, 7 days). Hwang et al^[29]^ also found a strong association between PM_2.5_ and COPD-related hospitalization on lags 0 to 5. However, Peel et al^[28]^ analyzed the relationship between PM_2.5_ and COPD-related ED visits on lags 0 to 2; Stieb et al^[27]^ only analyzed the impact of PM_2.5_ on COPD-related ED visits on lags 0 to 2. Seasonal and regional heterogeneity influence the health effects of PM. Peng et al^[30]^ collected data from 100 cities in the US and found a positive association between PM and mortality, especially in the summer in northeastern areas. There might be seasonal and regional variations in PM constituents,^[31]^ and different PM constituents may contribute to different health impacts.^[32]^ A meta-analysis review collected 12 studies and summarized that PM_2.5_ is positively correlated with COPD-related hospitalization and deaths.^[33]^ The present study’s findings also support the positive association between PM_2.5_ levels and COPD-related ED visits.

Our study showed that preschool asthma patients were more vulnerable to PM-related hospitalizations. Several studies have suggested that ambient air pollution, especially fine PM (PM_2.5_), is a modifiable risk factor for respiratory diseases among children.^[34, 35]^ Children may be more vulnerable to the health effects of ambient air pollution because of their higher rates of breathing, narrower airways, developing lungs, and frequent exposure to outdoor air.^[36]^ As mentioned earlier, PM_2.5_ is smaller in size and can penetrate deeper, and the chemical composition of PM_2.5_ contains more harmful substances.^[5]^ In our study, PM_2.5_ has a more substantial harmful health effect on asthma and COPD than PM_10_, suggesting that more attention should be paid to PM_2.5_. Increased PM_2.5_ and PM_10_ concentrations increased the number of respiratory disease patients in all age groups and increased the use of medical facilities. However, not everyone experiences the same effect. In asthma, health effects were more significant in pediatric patients under 5 years of age and in males. COPD was associated with greater health effects in elderly patients aged >65 years and in males. The health effects of increased concentrations of fine dust on patients with airway disease vary by sex, age, and disease. In Korea, the standard PM concentration is PM_10_ below 50 μg/m^3^ annual mean and PM_2.5_ below 15 μg/ m^3^ annual mean.^[37]^ In addition, the WHO concentration standard is PM_10_ below 20 μg/m^3^ annual mean and PM_2.5_ below 10 μg/m^3^ annual mean.^[38]^ The standard annual PM concentration in Seoul, Korea, is still considerably higher than that of the WHO. This study not only identified the effects of PM on patients suffering from respiratory diseases but also suggests the need for specialized standard concentrations of PM_10_ and PM_2.5_ to control air quality according to granular efforts by dividing them into sensitive categories considering accurate diagnosis, sex, and age.

This study is an indirect study based on the claims data of the National Health Insurance Service, and patients were selected based on claim codes rather than exact test results for diagnosis, such as pulmonary function test, and therefore the accuracy of the diagnosis may not have been optimal. However, we included the administration of medication as a requirement for disease diagnosis, in addition to clinical diagnosis, to overcome this limitation. The lack of analysis of indoor air quality management and its possible association with respiratory diseases is another limitation of this study. Considering the large proportion of time spent inside, further studies on indoor air quality management and the effects of PM_2.5_ and PM_10_ on respiratory patients are needed. Our study had several further limitations. First, the number of hospital visits was based on claims data associated with diagnoses of asthma and COPD defined according to the International Classification of Diseases codes, which may not reflect the patient’s condition during the hospital visit. Second, there was a lack of data on demographic factors, such as smoking history, lung function, dyspnea scale, and previous exacerbation history, which may influence hospitalizations of asthma and COPD patients. Finally, we could not account for the indoor air pollutants. Despite these limitations, our study also had a number of strengths, including the use of a time-stratified case-crossover design, which could minimize the effects of long-term seasonal trends, and the use of serial autocorrelation of the data to allow full adjustment for multiple confounding factors. We adjusted for the possible effects of seasonal and long-term trends in PM levels and hospitalization due to asthma or COPD. We also collected data to investigate the effects of PM on hospitalization on different lag days.

## 5. Conclusions

We found an association between PM and hospitalization due to asthma or COPD. Of the pollutants. Subgroup analysis showed that male asthma, preschool asthma, male COPD and senior COPD seniors had more hospitalizations. Besides, PM_2.5_ may have a more significant effect on airway disease patients than PM_10_. among the population of Seoul, South Korea. Therefore, patients with COPD and asthma should be cautioned against performing outdoor activities when PM levels, especially those of PM_2.5_, are high.

## Author contributions

All authors contributed conception, analysis, interpretation, revising, and final approval of the manuscript. CHH and HP served as a principal investigator and had full access to all of the data in the study. JHC take responsibility for the integrity of the data and the accuracy of the data analysis.
